# Caught you: threats to confidentiality due to the public release of large-scale genetic data sets

**DOI:** 10.1186/1472-6939-11-21

**Published:** 2010-12-29

**Authors:** Matthias Wjst

**Affiliations:** 1 Institute of Lung Biology and Disease, CPC - Comprehensive Pneumology Center, Helmholtz Center Munich - German Research Center for Environmental Health, Munich, Germany

## Abstract

**Background:**

Large-scale genetic data sets are frequently shared with other research groups and even released on the Internet to allow for secondary analysis. Study participants are usually not informed about such data sharing because data sets are assumed to be anonymous after stripping off personal identifiers.

**Discussion:**

The assumption of anonymity of genetic data sets, however, is tenuous because genetic data are intrinsically self-identifying. Two types of re-identification are possible: the "Netflix" type and the "profiling" type. The "Netflix" type needs another small genetic data set, usually with less than 100 SNPs but including a personal identifier. This second data set might originate from another clinical examination, a study of leftover samples or forensic testing. When merged to the primary, unidentified set it will re-identify all samples of that individual.

Even with no second data set at hand, a "profiling" strategy can be developed to extract as much information as possible from a sample collection. Starting with the identification of ethnic subgroups along with predictions of body characteristics and diseases, the asthma kids case as a real-life example is used to illustrate that approach.

**Summary:**

Depending on the degree of supplemental information, there is a good chance that at least a few individuals can be identified from an anonymized data set. Any re-identification, however, may potentially harm study participants because it will release individual genetic disease risks to the public.

## Background

Large-scale SNP data sets are shared with other research groups or even released on the Internet to foster new collaborations or to allow for second-look analysis [1], [2]. Study participants are not always informed about such data sharing because these kinds of data are assumed to be anonymous after stripping off personal identifiers like name or date of birth (a process also called pseudonymization).

## Discussion

This procedure, however, touches several aspects identified in the earlier literature such as confidentiality and genetic privacy [3], [4], [5].

Confidentiality has been seen in the past as a fundamental ethical principle in health care and breaching confidentiality is usually a reason for disciplinary action. It has been assigned such a great value because it directly originates from the patient's autonomy to control his or her own life. Releasing sensitive information in a professional patient-physician relationship is regarded by most patients as an implicit contract that doctors will keep all information confidential.

Such a contract is also inferred in genetic epidemiology [6,7]. The European Court of Human Rights therefore ruled in 2008 that the world's largest DNA database, based in the UK, violated Article 8 of the European Convention on Human Rights which protects privacy [8]. An earlier survey conducted by the Genetics and Public Policy Center found that the majority of the Americans surveyed supported genetic testing for research and health care, but 92% also felt concern that genetic test results revealing risk of future disease might be used in ways that would be harmful to a person [9]. Genetic privacy is therefore well founded in theory and appreciated in practice.

## Anonymity

Anonymity is derived from the Greek word ανωνυμία, meaning "no name", however it soon took on the meaning "non-identifiable". Instead of being truly "non-identifiable", anonymity may be seen as a variable level of hiding an identity that largely depends on surrounding information (statistically seen as a proposition of a true or false identity assignment with a Bayesian probability P for the proposition to be true or false). A set of banknotes in a pocket may be largely uninformative, as long as they do not contain a certain mix of issuing offices that will allow reconstruction of the travel history of the owner.

The assumption of anonymity of genetic data sets is tenuous if personal identifiers are merely removed and kept in another place [8]. Moreover, the usual k-anonymization strategy, where each relevant entity is hidden in at least k peers, is not feasible with such highly informative data sets [2]. Some authors even believe that de-identifying records is just a matter of economic investment ranging between $ 0 and $ 17,000 even for data protected under the "safe harbor" act, the U.S. Health Insurance Portability and Accountability Act [10].

Genetic data are intrinsically self-identifying [11], hence their use in forensic investigations. With the progress of genome-wide association studies, earlier work on genetic privacy has become outdated [3], [12], [13] and will finally be buried with the advent of whole genome sequencing [14]. Although current study leaflets still promise "strict confidentiality" to study participants, some authors conclude that anonymity is already a thing of the past [15]. Family history and genetic diagnoses are already being traced on the Internet [7] using dedicated websites [16].

## Attackers

Are there any real threats to anonymity? The answer may depend on the overall interest in re-identification of data driven by financial interests, (pseudo-) ethical reasons, personal interests or just curiosity. Benitez and Malin [10] describe three types of data intruders, notably prosecutor, journalist, and marketer types. The prosecutor attack has a specific target; the journalist attack is interested in identifying a particular record, while the adversary's goal in the marketer attack is to identify as many records as possible.

Heeney et al. [7] further examined the motivation of data intruders that range from scientists doing further research, police and secret services for forensic purposes, but also agents working in marketing, insurance or employment offices. In addition there is a large community interested in genealogy some with even "strong [...] motivations, including adoptees and donor-conceived children", who claim to have rights to de-identify genetic information.

There are already examples in the literature where a single individual could be identified [17]; surnames of individuals in the Hapmap samples could be traced [18] or James Watson's ApoE gene status inferred although not being released [19].

## Attack Types

At least two types of de-anonymization attacks can be differentiated. The first attack scenario comprises another rather small genetic data set of less than 100 SNPs but including personal identifiers (3). This second data set might originate from a later clinical examination, another study of leftover samples, or some forensic testing. This data set might then be merged to the large unidentified set by using identical markers in both sets. Although such a scenario is rather trivial from a technical point of view, it puts considerable pressure on tested individuals to avoid any retesting of their DNA (and of all close relatives). This type of attack may be called the "Netflix" type, named after the famous historical approach of two programmers who linked the anonymized Netflix video rental database with the true name Internet Movie Rating Database. They used a new class of statistical de-anonymization attack against a high-dimensional data set that included individual preferences, recommendations and transaction records [20].

The second type of a de-anonymization attack is a "profiling" approach that combines various levels of evidence. Although described earlier (for example as trail re-identification) this approach became only possible very recently. It employs different levels where information is being gathered and may be illustrated by a practical example using the "asthma kids" data set [21].

## Box

Genetic profiling using genome-wide SNP panels: A practical example.

(1) Background. The history of the sample collection is the most informative piece of information. A sample collection with origin "Munich" [21] already reduces a probability of an individual to be present in a data set from approximately 1:6,839 billion to 1:1,314 million. With the addition of the criteria "child" and "asthma", the probability is further reduced to 1:32.850. As study participation is not equal among social classes (with a bias of inner city participants and a bias of more severe cases attending a University department) the target group may be narrowed down to less than 1:2.000 and with known sex to less than 1:1.000.

(2) Subsets. Data subgroups and extreme dimensions of SNP array data may be checked by multidimensional scaling techniques. In the example above there is stratification in the data deposited online which is most likely explained by the inclusion of a minority group in the sample. Since public population databases include allele counts of hundreds of populations, this information can be used to further locate immigrant children to a region in Turkey. Even if there were would be no stratification but inbreeding, the inbreeding coefficient could also allow a guess about which population was included. Average linkage disequilibrium values may further tell if the population is more rural or more metropolitan. Pairwise identity by state distances between individuals allow to uncover also related individuals. Indeed, there are unreported siblings in the examined data set. Taking all this information together, this represents several unique cases that nurses and physicians at the Children' Hospital Munich may immediately recognize along with pharmacists, health insurance employees, school teachers or football coaches in inner city Munich.

(3) Phenotype prediction. A DNA-based prediction of individual phenotype characteristics is still in its infancy. So far, only the sex of a participant can be unequivocally determined using for example the AmelX/AmelY marker system; height prediction may be possible by using markers in hedgehog signalling or extracellular matrix genes; body mass can predicted by MC4R gene variants. Furthermore, we can guess skin and hair colour by OCA2 gene variants, while there might even be a small chance for an age prediction as aging introduces de novo mutations. Unique characteristics like refractive errors (MYP2), digital clubbing (HPGD), cryptorchidism (NR5A1), ear wax (ABCC11), bitter taste reception (TAS2R), freckling (BNC2), male baldness (AR, PAX1) or hair morphology (TCHH) is predictable together with behavioural traits like aggression (MAO-A) or anxiety type disorders (RGS2). There is a gene (AVPR1A) thought to influence divorce rate while alcohol dependency (GHS-R1A, NPY2R) and addictive smoking may be detectable as well (APBB1, CHRNA3). One gene became famous as the "god gene" (VMAT2) for being connected to religiosity, and another gene was assumed to influence intelligence (IQSEC2). Clearly, most of these predictions come with rather low predictive values, but it may be well be possible in the near future to run an SNP-derived prediction against a leaked Facebook profile.

(4) Disease prediction. Another source of information comes by scanning known disease variants. If present in a data set these variants are highly associated with the development of rare monogenic disease. Each of the ~2,400 OMIM phenotypes with known molecular background may be revealing when present in an individual DNA sample. In addition copy number variants as well as haplotypes may also be used for de-anonymization because these represent unique characteristic even in close relatives.

(5) Clinical status. Genotyping is usually done on a pool of blood cells while the composition of the pool may be affected by several diseases. For example cells undergoing somatic recombination may be detected in the pool if they are particularly high (leukaemia) or low (AIDS). There might be even a chance of finding foreign cells (microchimerism indicative of pregnancy or recent abortion) as even 1 in 1000 cells can be detected by genome-wide SNP arrays.

Depending on the data structure and the degree of supplemental information, there is a good chance that at least some individuals can be immediately de-identified by a profiling approach.

## Risks and defense

Any re-identification will expose individual genetic risks in the public as individuals become vulnerable to the consequences of genetic testing ranging from un-insurability, un-employability or other discrimination. It is difficult to anticipate any further use of genetic data while it is expected that threats to privacy and confidentiality will increase as genomic technologies are rolled out more widely.

Several options have been proposed on how to deal with genetic privacy in the future. These include open consent, better encoding techniques, or implementing legal constraints along with restricted data access.

Although open consent (no "promises of anonymity, privacy or confidentiality are made") acknowledges the fact that there are no anonymous genetic data, while agreeing to this may be only an option for individuals with "a master's degree in genetics" [2]. For genetic epidemiology studies, however, that rely on high response ratios, open consent is not a realistic option.

Better encoding could be another option. Making malicious data de-identification is, however, difficult to control on a worldwide scale while most countries still lack data protection laws. Also, more sophisticated encoding techniques are unlikely to prevent data misuse because they will just compete with better de-identification strategies.

The most feasible solution therefore will be highly restricted data access. Simply put - data not available cannot be decrypted. This policy seems to be adopted now by large research organizations like the NIH and the Wellcome Trust, who have already removed genetic data from their websites [6].

## Summary

Most importantly, it seems necessary to increase public awareness of genetic privacy and to inform probands continuously about the use of their samples and data [1]. The risks of re-identification of anonymized data should be included in informed consent procedures, and any data sharing needs to be explicitly approved by the DNA donor. As a measure of precaution, genetic data should not be distributed on public Internet sites, and data sets with more than 100 SNP markers should be removed from public web servers if not explicitly endorsed by the donor. As suggested before [11], data access should be restricted to scientific collaborations under confidentiality agreements only.

## List of abbreviations used

DNA: deoxyribonucleic acid; SNP: single nucleotide polymorphism; NIH: National Institute of Health; AIDS: acquired immune deficiency syndrome; MC4R, OCA2, MYP2, HPGD, NR5A1, ABCC11, TAS2R, BNC2, AR, PAX1, TCHH, MAO-A, RGS2, AVPR1A, GHS-R1A, NPY2R, APBB1, CHRNA3, VMAT2, IQSEC2 denote gene names: for details see http://www.genecards.org.

## Competing interests

The author declares that he has no competing interests.

## Authors' contributions

The author did all research and wrote the manuscript.

## Pre-publication history

The pre-publication history for this paper can be accessed here:

http://www.biomedcentral.com/1472-6939/11/21/prepub

## References

[B1] Mascalzoni D Hicks A Pramstaller P Wjst M Informed consent in the genomics era PLoS Med 2008 5 e192 10.1371/journal.pmed.0050192 18798689 PMC2535658

[B2] Lunshof JE Chadwick R Vorhaus DB Church GM From genetic privacy to open consent Nat Rev Genet 2008 9 406 411 10.1038/nrg2360 18379574

[B3] Lawrie G Genetic Privacy: a challenge to medico-legal norms 2002 Cambridge University Press

[B4] Lin Z Owen AB Altman RB Genetics. Genomic research and human subject privacy Science 2004 305 183 10.1126/science.1095019 15247459

[B5] Shepperd S Farndon P Grainge V Oliver S Parker M Perera R Bedford H Elliman D Kent A Rose P DISCERN-Genetics: quality criteria for information on genetic testing Eur J Hum Genet 2006 14 1179 1188 10.1038/sj.ejhg.5201701 16868557

[B6] Couzin J Genetic privacy. Whole-genome data not anonymous, challenging assumptions Science 2008 321 1278 10.1126/science.321.5894.1278 18772401

[B7] Heeney C Hawkins N de Vries J Boddington P Kaye J Assessing the Privacy Risks of Data Sharing in Genomics Public Health Genomics 2010 http://www.publichealth.ox.ac.uk/helex/publications/HeeneyEtAl2010 20339285 10.1159/000294150 PMC2872768

[B8] European Court Rules DNA Retention Illegal http://www.privacyinternational.org/article.shtml?cmd%5B347%5D=x-347-563175

[B9] U.S. Public Opinion on Uses of Genetic Information and Genetic Discrimination http://www.dnapolicy.org/resources/GINAPublic_Opinion_Genetic_Information_Discrimination.pdf

[B10] Benitez K Malin B Evaluating re-identification risks with respect to the HIPAA privacy rule J Am Med Inform Assoc 2010 17 169 177 10.1136/jamia.2009.000026 20190059 PMC3000773

[B11] Lowrance WW Collins FS Identifiablity in genomic research Science 2007 317 5838 600 602 10.1126/science.1147699 17673640

[B12] Rothstein MA Genetic secrets: protecting privacy and confidentiality in the genetic era 1997 New Haven: Yale University Press

[B13] Consortium P3G Church G Heeney C Hawkins N de Vries J Boddington P Kaye J Bobrow M Weir B Public access to genome-wide data: five views on balancing research with privacy and protection PLoS Genet 2009 5 e1000665 10.1371/journal.pgen.1000665 19798440 PMC2749921

[B14] Greenbaum D Du J Gerstein M Genomic anonymity: have we already lost it? Am J Bioeth 2008 8 71 74 10.1080/15265160802478560 19003717

[B15] Dolgin E Personalized investigation Nature Medicine 2010 16 953 955 10.1038/nm0910-953 20823867

[B16] Find biological family, missing family and family links http://www.dnareunion.org

[B17] Homer N Szelinger S Redman M Duggan D Tembe W Muehling J Pearson JV Stephan DA Nelson SF Craig DW Resolving individuals contributing trace amounts of DNA to highly complex mixtures using high-density SNP genotyping microarrays PLoS Genet 2008 4 e1000167 10.1371/journal.pgen.1000167 18769715 PMC2516199

[B18] Gitschier J Inferential genotyping of Y chromosomes in Latter-Day Saints founders and comparison to Utah samples in the HapMap project Am J Hum Genet 2009 84 251 258 10.1016/j.ajhg.2009.01.018 19215731 PMC2668019

[B19] Nyholt DR Yu CE Visscher PM On Jim Watson's APOE status: genetic information is hard to hide Eur J Hum Genet 2009 17 147 149 10.1038/ejhg.2008.198 18941475 PMC2986051

[B20] Narayanan AS How to break anonymity of the Netflix prize data set 2007 http://arxiv.org/abs/cs/0610105

[B21] Moffatt MF Kabesch M Liang L Dixon AL Strachan D Heath S Depner M von Berg A Bufe A Rietschel E Heinzmann A Simma B Frischer T Willis-Owen SA Wong KC Illig T Vogelberg C Weiland SK von Mutius E Abecasis GR Farrall M Gut IG Lathrop GM Cookson WOCM Genetic variants regulating ORMDL3 expression contribute to the risk of childhood asthma Nature 2007 448 470 473 10.1038/nature06014 17611496

